# MicroRNA-223 Expression Is Upregulated in Insulin Resistant Human Adipose Tissue

**DOI:** 10.1155/2015/943659

**Published:** 2015-07-27

**Authors:** Tung-Yueh Chuang, Hsiao-Li Wu, Chen-Chun Chen, Gloria Mabel Gamboa, Lawrence C. Layman, Michael P. Diamond, Ricardo Azziz, Yen-Hao Chen

**Affiliations:** ^1^Department of Obstetrics/Gynecology, Georgia Regents University, 1120 15th Street, Augusta, GA 30912, USA; ^2^Department of Biostatistics, Georgia Regents University, 1120 15th Street, Augusta, GA 30912, USA; ^3^Department of Surgery, Georgia Regents University, 1120 15th Street, Augusta, GA 30912, USA; ^4^Section of Reproductive Endocrinology, Infertility & Genetics, Georgia Regents University, 1120 15th Street, Augusta, GA 30912, USA; ^5^Institute of Molecular Medicine and Genetics, Georgia Regents University, 1120 15th Street, Augusta, GA 30912, USA; ^6^Neuroscience Program, Georgia Regents University, 1120 15th Street, Augusta, GA 30912, USA; ^7^Department of Medicine, Georgia Regents University, 1120 15th Street, Augusta, GA 30912, USA

## Abstract

MicroRNAs (miRNAs) are short noncoding RNAs involved in posttranscriptional regulation of gene expression and influence many cellular functions including glucose and lipid metabolism. We previously reported that adipose tissue (AT) from women with polycystic ovary syndrome (PCOS) or controls with insulin resistance (IR) revealed a differentially expressed microRNA (miRNA) profile, including upregulated miR-93 in PCOS patients and in non-PCOS women with IR. Overexpressed miR-93 directly inhibited glucose transporter isoform 4 (GLUT4) expression, thereby influencing glucose metabolism. We have now studied the role of miR-223, which is also abnormally expressed in the AT of IR subjects. Our data indicates that miR-223 is significantly overexpressed in the AT of IR women, regardless of whether they had PCOS or not. miR-223 expression in AT was positively correlated with HOMA-IR. Unlike what is reported in cardiomyocytes, overexpression of miR-223 in human differentiated adipocytes was associated with a reduction in GLUT4 protein content and insulin-stimulated glucose uptake. In addition, our data suggests miR-223 regulates GLUT4 expression by direct binding to its 3′ untranslated region (3′UTR). In conclusion, in AT miR-223 is an IR-related miRNA that may serve as a potential therapeutic target for the treatment of IR-related disorders.

## 1. Introduction

MicroRNAs (miRNAs) are short (20–24 nucleotide) noncoding RNAs involved in posttranscriptional regulation of gene expression. miRNA genes can be epigenetically regulated and miRNAs can themselves repress key enzymes that drive epigenetic remodeling and directly modulate gene transcription in the nucleus through recognition of specific target sites in promoter regions [1]. miRNAs influence many cellular functions including glucose and lipid metabolism [2–6]. Insulin resistant adipocytes are known to contain a differentially expressed miRNA profile [7]. In insulin resistant 3T3-L1 adipocytes, approximately 80 miRNAs have been found to be up- or downregulated [8], while miR-320 and miR-29 have been demonstrated to regulate insulin action through the PI3K/AKT pathway [5, 8].

Polycystic ovary syndrome (PCOS) is one of the most common endocrine disorders, affecting ≥7–9% of reproductive-aged women, even when defined conservatively [9]. About 60–70% of PCOS patients demonstrate insulin resistance (IR) above and beyond that predicted by body mass, race, or age, resulting in compensatory hyperinsulinemia and an increased risk for type 2 diabetes mellitus (T2DM) and metabolic syndrome. The underlying cellular mechanisms leading to IR in PCOS remain to be completely elucidated, as no gross defects in the traditional insulin signaling pathways have been found, including insulin binding, insulin receptor expression, and the IRS-1/PI3 K/AKT pathway [10, 11].

We previously reported that miR-93 is upregulated in adipose tissue (AT) from PCOS and non-PCOS women who display IR [11]. Overexpressed miR-93 directly inhibits glucose transporter isoform 4 (GLUT4) expression, influencing glucose metabolism. In addition we also observed that miR-223 was abnormally expressed in PCOS women with IR. miR-223 is overexpressed in insulin resistant myocardial cells and, paradoxically, overexpression of miR-223 by transfection has been reported to increase GLUT4 protein expression but not mRNA, thereby improving glucose uptake in cardiomyocytes [12].

It is unclear whether miR-223 may also regulate IR in adipocytes. In the present study, we examined the role of miR-223 in the AT of four groups of women: those without PCOS or IR; those without PCOS, but with IR; those with PCOS, but without IR; and women with PCOS and IR. We hypothesized that abnormal expression of miR-223 plays a role in the metabolic dysfunction of PCOS and IR.

## 2. Materials and Methods

### 2.1. Study Subjects

Subcutaneous abdominal AT samples from 33 women (30 White, 1 Black, and 2 Asian) were studied. Subjects were recruited at the Cedars-Sinai Medical Center in Los Angeles. The diagnosis of PCOS was performed as previously described [11]. In brief, the diagnosis of PCOS was made according to the National Institutes of Health (NIH) 1990 criteria [13]: (i) clinical evidence of hyperandrogenism and/or hyperandrogenemia; (ii) oligoovulation; and (iii) the exclusion of related disorders. Specific criteria for defining clinical hyperandrogenism, hyperandrogenemia, oligoovulation, and the exclusion of related disorders have been previously described [13]. All subjects had no significant illness including diabetes, had not received hormonal therapy or medications that could alter the metabolic or hormonal status for at least three months before the study, and were between the ages of 18 and 45 years. The study was approved by the Institutional Review Board, and all subjects gave informed written consent.

### 2.2. Hormonal Assays

Hormonal assays for total and free testosterone (T), dehydroepiandrosterone sulfate (DHEAS), insulin, and glucose were performed as previously described [14].

### 2.3. Adipose Tissue Biopsy and Real-Time PCR (qPCR)

Approximately 5 g of subcutaneous AT was excised through a small incision in the lower abdomen, as previously described (http://www.youtube.com/watch?v=Gy2pFUjDlDM [15]). Total RNA was extracted using the miRACLE Isolation Kit (Jinfiniti Biosciences, Augusta, GA). First-strand cDNAs of mRNA and miRNA were synthesized using the High Capacity cDNA Reverse Transcription Kit (Applied Biosystems, Foster City, CA) and First-Strand cDNA Synthesis Kit for miRNA (OriGene, Rockville, MD). Real-time PCR was performed using an iTaq Universal SYBR Green Supermix (Bio-Rad Laboratories, Inc., Hercules, CA). Primers were purchased from OriGene. Experiments were performed on an Applied Biosystems 7300 Real-Time PCR System.* ACTB* and miR-103 [16] were used as internal controls. Relative fold change of targets genes expression was calculated by using the 2^−ΔΔCt^ method.

### 2.4. Differentiation of Human Preadipocytes to Adipocytes

To induce differentiation, preadipocytes were cultured to full confluence and then maintained in differentiation medium (Cat# DM-2, Zen-Bio Inc., Research Triangle Park, NC) for one week (day 7 of differentiation) before being cultured in adipocyte medium (Cat# AM-1, Zen-Bio Inc., Research Triangle Park, NC) for an additional week (day 14 of differentiation).

### 2.5. Western Blot

For western blot analysis, 30 *μ*g protein was utilized. Blots were probed with specific primary antibodies and the appropriate secondary antibodies (Jackson ImmunoResearch Lab. West Grove, PA). *β*-actin was used as a loading control. GLUT4 antibody was purchased from Abcam, Cambridge, MA.

### 2.6. Transfection

Transfection was performed as described previously [17]. Briefly, for each well of 6-well plate, 2 *μ*g of plasmid was used for transfection. miR-223 overexpression plasmid (Cat# SC400292), noninsert empty plasmid control (Cat# PCMVMIR), and transfection reagent MegaTran 1.0 (Cat# TT200002) were purchased from OriGene (Rockville, MD). Assays were done at 48 hours after transfection.

### 2.7. Luciferase Reporter Assay

A luciferase reporter assay was performed as described previously [17]. GLUT4 3′UTR luciferase plasmid was purchased from OriGene (Rockville, MD). The 3′UTR luciferase plasmid (1 *μ*g) with either miR-223 overexpression or empty plasmid (2 *μ*g) was transfected in each well of a 12-well plate. 48 hours after transfection luciferase activity was assayed by a luciferase assay system (Promega, Madison, WI) measured on a fluorescence microplate reader (POLARstar Omega, BMG Labtech, Germany).

### 2.8. Glucose Uptake Assay

6-NBDG (6-(N-(7-nitrobenz-2-oxa-1,3-diazol-4-yl)amino)-6-deoxyglucose; Life Technologies, Carlsbad, CA) was used to determine insulin-stimulated glucose uptake in human differentiated adipocytes. Briefly, insulin-stimulated glucose uptake was determined by first changing the adipocyte medium to a low-glucose serum free (LGSF) medium (0.1% BSA) for 4 hours. Insulin (100 nM) was added and incubated for another 1 hour. After incubation, 6-NBDG (20 *μ*M) was added and incubated for 40 minutes. After incubation with 6-NBDG, adipocytes were washed three times with PBS, lysed by adding lysis buffer. 6-NBDG in cell lysate was measured on a fluorescence microplate reader (POLARstar Omega, BMG Labtech, Germany; excitation: 485 nm, emission: 535 nm).

### 2.9. Statistical Analysis

Insulin resistance at baseline was estimated using the homeostasis model assessment (HOMA-IR); a HOMA-IR value <2.5 was considered normal and a HOMA-IR value ≥2.5 indicated IR [18]. Comparisons of multiple groups were carried out by ANOVA followed by a posttest analysis using the Fisher (among groups) and Dunnett (compared to controls) tests (XLSTAT Software, NY). Logistic regression was used to adjust data for body mass index (BMI). Group comparisons (PCOS versus non-PCOS and IR versus non-IR) were carried out by unpaired Student's *t*-test (SAS 9.3, SAS Institute Inc., Cary, NC). Significant differences were defined as *P* < 0.05. All values are presented as mean ± SEM.

## 3. Results

Of the 33 subjects included, 15 (7 without and 8 with PCOS) did not have IR as defined. Among the 18 subjects with IR, 8 did not have PCOS and 10 had PCOS.  Table 1 depicts the clinical characteristics of the subjects included. As expected, subjects with PCOS had higher values for terminal body and facial hair growth and free T; and subjects deemed to have IR by HOMA-IR also had higher insulin levels than those without IR. While no subjects had diabetes as measured by fasting glucose, mean glucose in women with PCOS and IR was higher than non-PCOS non-IR women.

**Figure 1:**
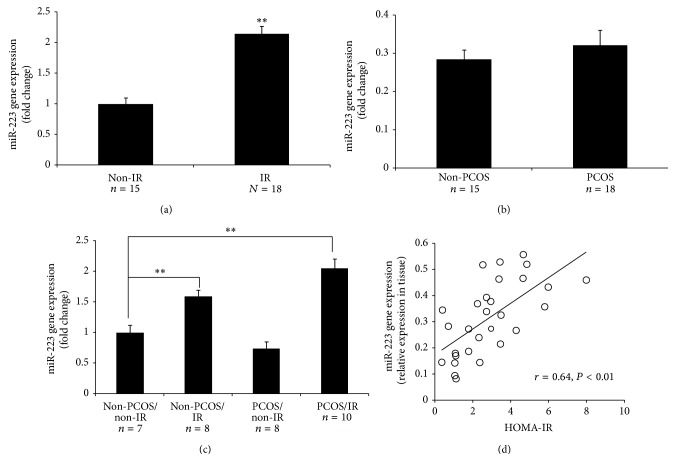
miR-223 expression in human AT. (a) Depicted is comparison of the levels of miR-223 expression in the AT of women with and without IR; miR-223 was significantly overexpressed in the IR group. (b) Depicted is comparison of the levels of miR-223 expression in the AT of women with and without PCOS; the expression of miR-223 did not differ between two groups. (c) Depicted is comparison of the levels of miR-223 expression in the AT of women: (A) without PCOS or IR, (B) without PCOS (non-PCOS), but with IR, (C) with PCOS and IR, and (D) with PCOS, but without IR; the expression of miR-223 was significantly higher in the AT in women with IR. ^∗∗^ *P* < 0.01 comparing tissues from women with or without PCOS and with IR versus that of women without PCOS or IR. (d) Depicted is the association between miR-223 expression and HOMA-IR. (Two group comparisons, (a) and (b), were carried out by unpaired Student's *t*-test; comparison of (d) was carried out by ANOVA followed by a posttest by using the Fisher (among groups) and Dunnett (compared to control group) test.)

The expression of miR-223 was significantly increased among all women with IR (*P* = 0.0004; Figure 1(a)). However, no difference in miR-223 expression was detected (Figure 1(b)) with regard to PCOS status. Comparing all four subgroups (7 subjects without PCOS and without IR, 8 without PCOS but with IR, 8 with PCOS but without IR, and 10 with both PCOS and IR), miR-223 was only significantly overexpressed in the two groups of women with IR, compared to subjects without PCOS and without IR (*P* < 0.01; Figure 1(c)). Next, we examined the association of miR-223 expression with measures of IR, including HOMA-IR. Our results indicated that miR-223 expression was positively correlated with HOMA-IR (*r* = 0.64, *P* < 0.01; Figure 1(d))

Subjects with IR (regardless of the presence of PCOS) tended to have a greater mean body mass index (BMI) than subjects without IR (Table 1), a difference that reached significance only in women without PCOS, between those with IR and those without IR. There were no statistical differences in age. To gauge the possible effects of these differences on miR-223 expression we first determined whether an association existed between miR-223 expression and age or BMI for the entire group combined. Our results indicate that miR-223 expression did not vary according to age (*r* = −0.11, *P* = 0.288) but was positively correlated with BMI (*r* = 0.46, *P* = 0.01). Consequently, we compared miR-223 expression values adjusted for BMI for subjects with and without PCOS and with and without IR. The adjustment did not change our results, with the difference in miR-223 expression between women with and without IR (*P* = 0.0193) and the absence of a difference between women with and without PCOS (*P* = 0.1178) (Table 2) remaining.

**Figure 2:**
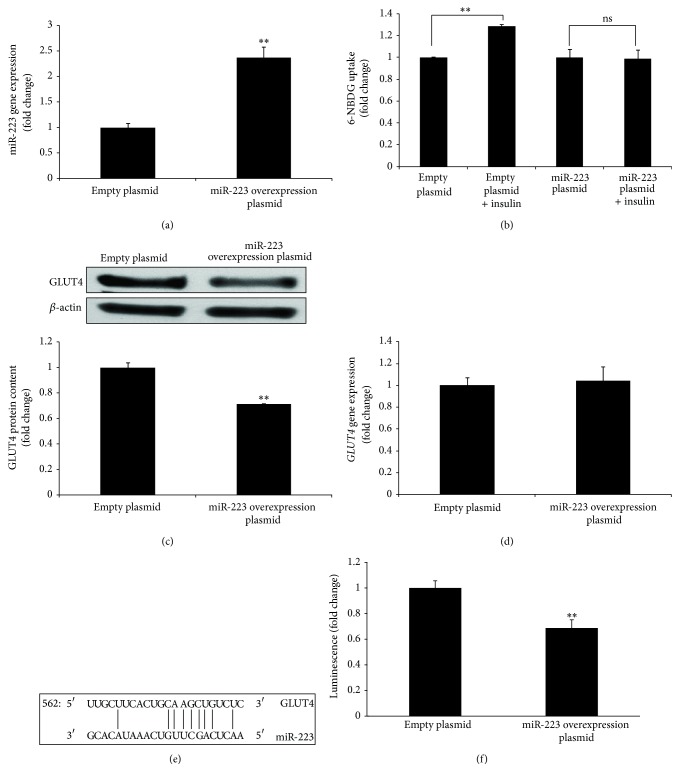
miR-223 regulates GLUT4 expression in human differentiated adipocytes. (a), (c), and (d) Depicted are the miR-223, GLUT4 protein, and* GLUT4* gene expression in human differentiated adipocytes after being transfected with miR-223 overexpression plasmid. (b) Depicted is 6-NBDG uptake stimulated by insulin in human differentiated adipocytes after being transfected with miR-223 overexpression plasmid. (e) Depicted is a GLUT4 3′UTR predicted binding site for miR-223. (f) Depicted is the GLUT4 3′UTR luciferase reporter which was repressed by overexpression of miR-223. ^∗∗^ *P* < 0.01 comparing to empty plasmid. (In (a), (c), (d), and (f), the comparisons of significance were carried out by unpaired Student's *t*-test. In (b), the comparison of significance was carried out by ANOVA followed by a posttest by using the Fisher (among groups) and Dunnett (compared to control group) test.)

To determine the role of and mechanism by which miR-223 induced IR in adipocytes, we overexpressed miR-223 in human differentiated adipocytes* in vitro* to achieve an approximately twofold increase in expression compared to empty plasmid controls (*P* < 0.01; Figure 2(a)), similar to the level of miR-223 overexpression in human AT (Figure 1(a)). We found overexpression of miR-223 inhibited glucose uptake stimulated by insulin in human differentiated adipocytes* in vitro* (Figure 2(b)). In addition, our data indicated that the induced overexpression of miR-223 was associated with a decrease in GLUT4 protein content (Figure 2(c)), but not* GLUT4* gene expression (Figure 2(d)). These data suggest GLUT4 could be a direct target of miR-223. Analysis of GLUT4 3′UTR sequence using the free energy-based miRNA prediction program PITA [18] revealed one potential target site for miR-223 in GLUT4 (Figure 2(e)). To address whether direct binding of miR-223 to the GLUT4 3′UTR is responsible for the observed suppression of GLUT4, we performed a luciferase assay in which direct binding of miR-223 to the vector GLUT4 3′UTR gene transcript would repress a luciferase reporter. Transient cotransfection of miR-223 and luciferase expression plasmids in human differentiated adipocytes demonstrated direct binding of miR-223 to the GLUT4 3′UTR site, resulting in a significant reduction in luciferase (Figure 2(f)).

**Figure 3:**
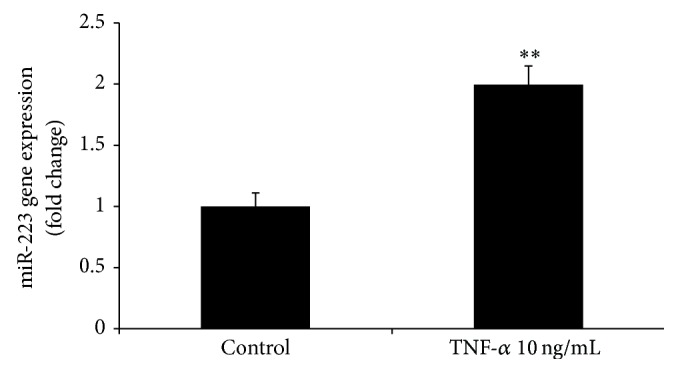
TNF-*α* induces expression of miR-223 in human differentiated adipocytes. Human differentiated adipocytes were treated with TNF-*α* (10 ng/mL) for 24 hours. Expression of miR-223 was detected by real-time PCR. ^∗∗^ *P* < 0.01 comparing to control; the comparisons of significance were carried out by unpaired Student's *t*-test.

As tumor necrosis factor-*α* (TNF-*α*) induces IR in adipocytes [19], the role of TNF-*α* in the regulation of miR-223 expression in human differentiated adipocytes was also examined. Consistent with the hypothesis that TNF-*α* increases IR in adipocytes, at least in part, via the modulation of miR-223 expression, we observed that treatment of human differentiated adipocytes with TNF-*α* (10 ng/mL) for 24 hours significantly increased miR-223 expression (Figure 3).

## 4. Discussion

In a previous study, we examined the expression of miR-223 in AT from a total of 25 subjects, and our findings indicated that miR-223 tended to be overexpressed in PCOS and non-PCOS women with IR [11]. These trends were confirmed in the present study, analyzing a larger number of subjects, such that miR-223 was significantly overexpressed in women with IR, regardless of PCOS status.

GLUT4 is the major protein responsible for insulin-mediated glucose translocation into adipocytes [20] and plays an important role in the regulation of glucose homeostasis. In adipocytes a 50% decrease in GLUT4 content leads to a 50% decrease in GLUT4 translocation [21]. Moreover, AT-specific GLUT4 impacts glucose tolerance, insulin sensitivity, and glucose metabolism* in vivo* [22, 23].* GLUT4* gene expression in AT correlated with HOMA-IR [11]. In cardiomyocytes, overexpression of miR-223 stimulates glucose uptake and increases GLUT4 protein content but not the level of mRNA [12].

In the present study, we examined the regulation of GLUT4 expression by miR-223. Similar to cardiomyocytes [12], overexpression of miR-223 in adipocytes did not alter GLUT4 mRNA expression. However, unlike cardiomyocytes, miR-223 overexpression was associated with a decrease in GLUT4 protein content and glucose uptake in AT. The discrepancy between cardiomyocytes and adipocytes could solely reflect differences in tissue specific regulation. However, miR-223 does appear to be overexpressed in AT and the myocardium of IR subjects and suggests that miR-223 may serve as a therapeutic target for IR.

In cardiomyocytes, overexpression of miR-223 enhances insulin-stimulated glucose uptake by increasing GLUT4 but not by altering insulin signaling and AMPK activity (baseline and phosphorylation) [12]. Insulin signaling components and AMPK are not targets of miR-223. This suggests that reduced insulin-stimulated glucose uptake in miR-223 overexpressed human differentiated adipocytes could be due to decreased levels of GLUT4, not by altering GLUT4 translocation.

That miR-223 decreased GLUT4 protein, but not mRNA, in adipocytes which suggests that miR-223 may regulate GLUT4 expression by binding to its 3′UTR. Although* in silico* analysis (algorithms miRanda, PicTar, and TargetScan) indicated that GLUT4 was not a predicted target of miR-223, we found one potential binding site in the 3′UTR sequence of GLUT4 by using the free energy-based miRNA prediction program PITA. Furthermore, by the GLUT4 3′UTR reporter assay, we demonstrated that miR-223 regulates GLUT4 expression by direct binding to its 3′UTR site.

We previously reported that the expression of miR-93 was significantly increased in the subcutaneous abdominal AT of all PCOS patients studied and non-PCOS women with IR [11]. Alternatively, miR-223 was increased in women with IR, regardless of PCOS status. Both miR-93 and miR-223 regulated GLUT4 protein content in adipocytes [11]. Together, these data suggest that miR-93 expression is associated with both IR and PCOS, whereas miR-223 is not involved in PCOS per se but is related to IR. As we previously noted that PCOS women with IR had the lowest expression of GLUT4 [11], it is possible that miR-93 and miR-223 may have additive effects on the regulation of GLUT4 expression.

In addition to our findings that miR-223 and miR-93 regulate IR in AT by targeting GLUT4, these two miRNAs have also been found to suppress proinflammatory activation of macrophages by targeting IRAK4 (for miR-93) [24] and Pknox1 (for miR-223) [25]. Macrophage activation is associated with IR [26]; therefore, these data suggest that miR-223 and miR-93 could also regulate IR by regulating inflammation.

miR-223 and miR-93 have been found to have similar functions yet they may or may not target the same genes. Both miRNAs regulate cancer activity by targeting the same gene E2F1 [27, 28]. However, to promote cancer activity, they also target different genes including C/EBP*β*, FOXO1, NFI-A, STAT5A, ARTN, FBXW7, and SEPT6 (for miR-223) [29–33] and FUS1, RhoC, PTEN, CDKN1A, TGF*β*R2, and NRF2 (for miR-93) [34–38]. Both miRNAs act as antiangiogenesis regulators, but miR-93 directly targets vascular endothelial growth factor A (VEGF-A) [39], while miR-223 targets *β*1 Integrin [40]. These data suggest that miR-223 and miR-93 may also have additive effects on these functions.

In conclusion, our data indicates that miR-223 is overexpressed in the subcutaneous AT of subjects with IR, regardless of PCOS status, and that miR-223 expression is positively associated with IR* in vivo*. Overexpression of miR-223 decreased GLUT4 protein content and inhibited insulin-stimulated glucose uptake in cultured human adipocytes. In addition TNF-*α* induced miR-223 expression* in vitro*, suggesting that TNF-*α* exerts its negative effect on insulin action at least in part through its modulation of the expression of this miRNA. Together these data suggest the possibility that miR-223 could be a potential therapeutic target for IR.

## Figures and Tables

**Table 1:** Clinical characteristics of study subjects.

	Non-PCOS without IR (*n* = 7)	Non-PCOS with IR (*n* = 9)	PCOS (*n* = 8)	PCOS with IR (*n* = 11)
BMI (kg/m^2^)	24.58 ± 5.47	34.97 ± 6.76^∗∗^	24.81 ± 3.16	31.31 ± 5.55
Age (yrs)	33.17 ± 8.47	34.86 ± 7.08	28.29 ± 2.36	27.00 ± 4.76
mFG score	0.17 ± 0.41	0.63 ± 0.92	5.71 ± 5.40^∗^	7.00 ± 3.59^∗∗^
Free testosterone (pg/mL)	1.73 ± 0.33	2.66 ± 1.29	4.56 ± 2.47^∗^	6.66 ± 2.51^∗∗^
Total testosterone (ng/mL)	26.50 ± 5.54	23.86 ± 3.76	45.71 ± 25.42	37.90 ± 19.14
DHEAS (*µ*g/dL)	127.8 ± 30.7	252.7 ± 31.7^∗^	286.3 ± 31.2^∗∗^	244.6 ± 23.1^∗^
Fasting glucose (mcg/dL)	68.50 ± 14.39	87.38 ± 8.85	88.14 ± 5.58^∗^	93.15 ± 17.03^∗∗^
Fasting insulin (mIU/mL)	5.00 ± 3.56	13.75 ± 3.24^∗^	5.50 ± 2.35	20.43 ± 7.16^∗∗++^
HOMA-IR	0.88 ± 0.67	2.94 ± 0.66^∗^	1.20 ± 0.58	4.65 ± 1.61^∗∗++^
Prolactin (ng/mL)	13.3 ± 5.50	10.99 ± 5.54	10.37 ± 5.14	12.83 ± 5.99
TSH (IU/mL)	2.05 ± 0.53	2.94 ± 1.48	1.66 ± 0.70	1.95 ± 0.93
17-HP (ng/dL)	25.29 ± 9.23	21.14 ± 7.24	29.90 ± 20.09	35.86 ± 14.86

Data are expressed as mean ± SD.

^∗∗^ *P* < 0.01 versus control group.

^∗^ *P* < 0.05 versus control group.

^++^ *P* < 0.01 versus PCOS group.

mFG score is the modified Ferriman-Gallwey hirsutism score; HOMA-IR is homeostasis model assessment for estimating insulin resistance. DHEAS is dehydroepiandrosterone sulfate, TSH is thyroid stimulating hormone, and 17-HP is 17 alpha-hydroxyprogesterone.

**Table 2:** Relationship of miR-223 to PCOS and IR status and BMI.

	Unadjusted			Adjusted		
	Odds ratio	95% CL^∗∗^ (lower, upper)	*P* value	Odds ratio	95% CL^∗∗^ (lower, upper)	*P* value
IR versus non-IR						
miR-223^∗^	1.144	(1.037, 1.262)	0.0071	1.134	(1.021, 1.260)	0.0193
BMI	1.135	(0.985, 1.308)	0.0810	1.029	(0.881, 1.202)	0.7158

	Unadjusted			Adjusted		
	Odds ratio	95% CL^∗∗^ (lower, upper)	*P* value	Odds ratio	95% CL^∗∗^ (lower, upper)	*P* value

PCOS versus non-PCOS						
miR-223^∗^	1.027	(0.971, 1.087)	0.3525	1.059	(0.985, 1.139)	0.1178
BMI	0.929	(0.822, 1.049)	0.2350	0.875	(0.749, 1.023)	0.0937

^∗^The miR-223 has been multiplied by 100 for the depiction of the odds ratio and confidence limit; the *P* value of the comparisons is not affected.

^∗∗^95% CL is the 95% confidence limit.
